# Camera Calibration with Weighted Direct Linear Transformation and Anisotropic Uncertainties of Image Control Points

**DOI:** 10.3390/s20041175

**Published:** 2020-02-20

**Authors:** Francesco Barone, Marco Marrazzo, Claudio J. Oton

**Affiliations:** 1 Scuola Superiore Sant’Anna, TeCIP Institute, Via Giuseppe Moruzzi 1, 56127 Pisa, Italy; c.oton@santannapisa.it; 2 Baker Hughes, Via Felice Matteucci 2, 50127 Florence, Italy

**Keywords:** camera calibration, DLT, PnP, weighted DLT, uncertainty, covariance, robustness

## Abstract

Camera calibration is a crucial step for computer vision in many applications. For example, adequate calibration is required in infrared thermography inside gas turbines for blade temperature measurements, for associating each pixel with the corresponding point on the blade 3D model. The blade has to be used as the calibration frame, but it is always only partially visible, and thus, there are few control points. We propose and test a method that exploits the anisotropic uncertainty of the control points and improves the calibration in conditions where the number of control points is limited. Assuming a bivariate Gaussian 2D distribution of the position error of each control point, we set uncertainty areas of control points’ position, which are ellipses (with specific axis lengths and rotations) within which the control points are supposed to be. We use these ellipses to set a weight matrix to be used in a weighted Direct Linear Transformation (wDLT). We present the mathematical formalism for this modified calibration algorithm, and we apply it to calibrate a camera from a picture of a well known object in different situations, comparing its performance to the standard DLT method, showing that the wDLT algorithm provides a more robust and precise solution. We finally discuss the quantitative improvements of the algorithm by varying the modules of random deviations in control points’ positions and with partial occlusion of the object.

## 1. Introduction

Many computer vision applications, such as robotics, photogrammetry, or augmented reality, require camera calibration algorithms. The calibration is the estimation of the parameters of the camera model from given photos and videos, acquired with the camera. Camera parameters are both extrinsic and intrinsic: extrinsic parameters are pose dependent, while the intrinsic ones are related to the intrinsic properties of the camera itself.

A camera model is a mathematical description of the projection of a 3D point in the real world on the 2D image plane. The pinhole camera model assumes no distortion and a small aperture size; thus, the projection is linear, and it is fully described by a 3×4 projection matrix (or camera matrix). Control points are usually used for the parameters’ estimation. They are points whose coordinates are known both in the 3D real world and in the 2D image plane. How the control points are chosen influences the estimation of parameters: a poor choice of control points requires a robust estimation algorithm. The most common procedures exploit a photo of an object whose geometry and pose in space are known. A chessboard pattern is usually used because corners in the chessboard pattern are very easy to identify and its geometry is simple.

When such a pattern is not available, auto-calibration algorithms could be used: they require multiple images to extract information to cope with the lack of a known object in the scene. In this case, features from multiple frames of a moving target (or moving camera) have to be extracted, then the correspondences between features of the different images have to be found [1,2]. Several video frames could be used for accurate auto-calibration procedures, especially offline when there are no constraints on execution times [3]. Plane surfaces are also commonly found in a scene, and some works use them to calibrate cameras [4,5].

Some calibration procedures aim for a one-off estimation of the intrinsic parameters of the camera, which will not vary in time. Once the intrinsic parameters are known, an online algorithm has to estimate only the camera pose. Given a set of *n* control points and given the intrinsic parameters, the estimation of the camera pose is called the Perspective-*n*-Points problem (P*n*P). Three is the smallest number of correspondences that yields a finite number of solutions [6,7]. P*n*P with three correspondences points is called P3P.

There are many different solutions to P*n*P [8,9], and some of them are very efficient with an O(n) complexity [10,11]. Other algorithms also provide some internal parameters, such as the focal length [12,13]. The sensitivity to the control points is critical. In particular, the RANSAC algorithm [14] became very popular because of its robustness to outliers. A recent algorithm developed by Ferraz (EPP*n*P [15]) reaches even better performance and higher robustness. He also integrated the uncertainties of features point [16], given the internal parameters.

If intrinsic parameters are unknown, a complete calibration is required. Many analytical methods were developed in the past especially by photogrammetrists [17]. The most known is the Direct Linear Transformation (DLT [18]) algorithm, which can compute the projection matrix from at least six control points. If there are more than six points, DLT minimizes the least squared error (LSQ) of reprojected points. Then, from the projection matrix, it is possible to extract the parameters of the pinhole camera model [2]. Because of LSQ, DLT is not robust to outliers [19]. To reduce gross errors, Molnár [20] used the Huber estimator, based on a weight function that limits the accounting of errors for outliers. Uncertainties were also integrated in the EPP*n*P ([15]) algorithm to solve the P*n*P problem, but it assumed that the internal parameters were known [16].

Current research for camera calibration faces many different problems, such as: automatic calibration methods [21], multiple cameras’ calibration [22], and distortion parameters’ estimation. For non-linear cameras’ calibration, Bouguet developed a general method, which included radial and tangential distortion [23], while Mei extended the algorithm also to omnidirectional cameras by means of multiple views of a planar pattern [24]. Further advances for omnidirectional camera calibration were done by Scaramuzza, who developed and implemented a fully automatic and iterative procedure for non-linear camera calibration [25,26].

Compared to DLT, non-linear camera models have the disadvantage of needing multiple images to estimate the distortion parameters; indeed, trying to estimate many parameters from only few points of a single image leads to an ill conditioned problem. A large set of control points is available especially when calibration patterns are used and corners are easy to detect. In particular applications, the number of control points is required to be low. For example, even if calibration patterns for an infrared camera exist [27], they cannot be used for an infrared camera inside a gas turbine.

Temperature mapping of gas turbine rotor blades is extremely important both for condition health monitoring and blade design optimization. Infrared thermography allows contactless measurements of the rotor blade surface temperature in gas turbines [28], but the mapping between the 2D image and the 3D blade requires adequate camera calibration. Thus, the blade itself has to be used as the calibration frame: indeed, its geometry is well known, and usually, an accurate 3D model is available. Nevertheless, the blade is always partially in the field of view.

Moreover, camera lenses are subject to high temperature variation and the intrinsic parameters, such as the focal length changes with temperature; thus, offline calibration of intrinsic parameters cannot be done.

Manual calibration is required because infrared images of blades usually have low quality and low resolution; thus, images have a limited number of features (such as corners and edges), and the number of control point is very limited.

To solve this problem, in this paper, we propose a method for camera calibration suitable for a low number (less than 10) of control points with anisotropic uncertainty. Assuming a different bivariate Gaussian distribution of the position error of each control point, its anisotropic uncertainty is determined by the uncertainty ellipse, within which the control point is estimated to be. The proposed method, based on direct linear transformation, consists of minimizing the weighted reprojection error of control points. The weight matrix is chosen according to the axis lengths and axis rotation of the uncertainty ellipses. Because of that, the presented weighted variation of the DLT (wDLT) includes the uncertainty information, improving the average accuracy, especially with few control points.

The wDLT algorithm we propose requires a photo of a well known 3D object (such as the rotor blade in a gas turbine).

Uncertainty ellipses could also be used to constrain a point along the direction of an edge, or a segment: in this case, the uncertainty is high along the edge, while it is low in the direction perpendicular to it. Exploiting the uncertainty becomes necessary when there are only a few control points, and some of them are almost exact, while the other ones are approximated.

In the following sections, we show the DLT algorithm for camera calibration and how to set the weight matrix for wDLT according to the uncertainty areas of control points. Then, we compare the performance of three algorithms (DLT, Bouguet’s method [23], and wDLT) using the reprojection error on 21 real images with only seven control points each. We evaluate the robustness of the algorithms, introducing fictitious errors to control points. Infrared images of rotor blades inside gas turbine always have a partial view of the blade itself, because of small spaces and the design constraint of the camera system; thus, we also simulate different scenarios where the object is partially occluded, making it difficult to find the control points manually, leading to an accuracy error. We also evaluate the sensitivity of uncertainty values. Results show that wDLT reaches higher average accuracy when the control points are perturbed with fictitious errors, and it allows camera calibration in some evaluated scenarios where the standard DLT and Bouguet’s method fail.

## 2. Materials and Methods

In this section, we first describe the weighted direct linear transformation algorithm and how we propose to choose the weight matrix; then, we describe the tests we performed on real images. The implementations of the DLT and wDLT algorithms were realized with MATLAB R2018b [29]; Bouguet’s method was performed by using the functions and scripts of the Camera Calibration toolbox for MATLAB [23].

### 2.1. The Weighted Direct Linear Transformation Algorithm

Considering the pinhole approximation, the camera model maps the 3D world points to 2D image points through a linear projection operation, which is expressed by the following relation:

(1) $$\lambda {\hat{x}}_{i}={\mathrm{PX}}_{i}$$

where $X_{i}={\left[X_{i},Y_{i},Z_{i},1\right]}^{T}$ is the *i*th world point in homogeneous coordinates, ${\hat{x}}_{i}={\left[x_{i},y_{i},z_{i}\right]}^{T}$ is a 3D representation of the ith projected point, $P\in {ℜ}^{3\times 4}$ is the projection matrix, and λ∈ℜ is a free parameter. The parameter λ is usually set equal to the reciprocal of $z_{i}$, such as ${\tilde{x}}_{i}=\lambda {\hat{x}}_{i}=[u_{i}$ $v_{i}$ ${1]}^{T}$ becomes the ith image point in the homogeneous coordinate. Applying the DLT algorithm (see Appendix A), for each correspondence between $X_{i}$ and ${\tilde{x}}_{i}$, two equations could be written:

(2) $$\left[\begin{array}{ccc} -X_{i}^{T} & 0^{T} & u_{i}X_{i}^{T}\\ 0^{T} & -X_{i}^{T} & v_{i}X_{i}^{T} \end{array}\right]\left[\begin{array}{c} p_{1}^{T}\\ p_{2}^{T}\\ p_{3}^{T} \end{array}\right]=M_{i}p=\left[\begin{array}{c} 0\\ 0 \end{array}\right]$$

where $p_{1}$, $p_{2}$, and $p_{3}$ are the rows of the projection matrix P, which have to be determined. Writing the equations for Ncorrespondences, the following equation system is obtained:

(3) $$\mathrm{Mp}=\left[\begin{array}{c} M_{1}\\ \vdots \\ M_{N} \end{array}\right]\left[\begin{array}{c} p_{1}^{T}\\ p_{2}^{T}\\ p_{3}^{T} \end{array}\right]=\left[\begin{array}{c} 0\\ \vdots \\ 0 \end{array}\right]$$

Considering that p is a 12 element array and each correspondence adds two equations, at least six equations are needed. Thus, for N≥6, an LSQ solution of the homogeneous linear system Mp=0 is the eigenvector of $M^{T}M$ corresponding to the minimum eigenvalue.

#### 2.1.1. Weights of the Equations

The proposed approach is based on modeling the error on the image plane as a bivariate Gaussian distribution, such as Ferraz et al. [16] did for the PnP problem. The covariance matrix $C_{i}$ of the error distribution could be written as follows:

(4) $$C_{i}=\left[\begin{array}{cc} \sigma_{x_{i}}^{2} & 0\\ 0 & \sigma_{y_{i}}^{2} \end{array}\right]R\left(\theta_{i}\right)$$

where $R\left(\theta_{i}\right)\in {ℜ}^{2\times 2}$ is the rotation matrix that rotates an angle $\theta_{i}$ and $\sigma_{x_{i}}$ and $\sigma_{y_{i}}$ are the standard deviations along the principal axes.

The weight matrix is defined by the following equation:

(5) $$W_{i}=\left[\begin{array}{cc} 1/\sigma_{x_{i}} & 0\\ 0 & 1/\sigma_{y_{i}} \end{array}\right]R\left(\theta_{i}\right)$$

Each equation of (3) is left multiplied by a weight matrix $W_{i}$ that depends on the covariance matrix of the expected error distribution for the ith point. This yields the following equation:

(6) $$\hat{M}p=\mathrm{WMp}=\left[\begin{array}{c} W_{1}M_{1}\\ \vdots \\ W_{N}M_{N} \end{array}\right]\left[\begin{array}{c} p_{1}^{T}\\ p_{2}^{T}\\ p_{3}^{T} \end{array}\right]=\left[\begin{array}{c} 0\\ \vdots \\ 0 \end{array}\right]$$

where W is a diagonal block matrix whose blocks on the diagonal are $W_{i}$.

The LSQ solution of the homogeneous linear system in Equation (6) is the eigenvector of ${\hat{M}}^{T}\hat{M}$ corresponding to the minimum eigenvalue. The error to minimize is weighted by the matrix W, so that for the ith point, the reprojection error along the axis $x_{i}$ (or $y_{i}$), which is tilted by an angle $\theta_{i}$ with respect to the horizontal (or vertical) image axis, is accounted for less if the $\sigma_{x_{i}}$ (or is $\sigma_{y_{i}}$) is high and vice versa.

#### 2.1.2. Covariance Matrix Estimation

The error is considered to be a bivariate Gaussian distribution, whose average is the ith control point and covariance matrix is $C_{i}$. Here, the estimation of $C_{i}$ is done by the manual assessment of the confidence region, the region within which each point is almost surely. For a bivariate distribution, this region is an ellipse. This ellipse is fully determined by the length $l_{x_{i}}$ and $l_{y_{i}}$ of the two orthogonal principal axis and the direction $\theta_{i}$ of $x_{i}$ axes. The standard deviation of the bivariate Gaussian distribution is set to a third of the length of the axis: $\sigma_{x_{i}}=l_{x_{i}}/3$ and $\sigma_{y_{i}}=l_{y_{i}}/3$. Thus, the covariance matrix is computed using Equation (4). The rotation matrix $R(\theta_{i})$ aligns the image axis to the axis of the confidence ellipse of the ith point. For our purposes, it is not the absolute value of $\sigma_{x_{i}}$ and $\sigma_{y_{i}}$ that is relevant, but their ratio.

### 2.2. Tests on Real Images

The following tests aimed to compare the wDLT performance with the standard DLT and Bouguet’s method (BOU), highlighting the advantages of using uncertainty information when the number of control points is low. The approach would reach similar results if the uncertainties of each point were equal (i.e., if the covariance matrix $C_{i}$ was the same scalar matrix for each i) or if the number of points was equal to six, that is the least needed for both algorithms.

**Figure 1:**
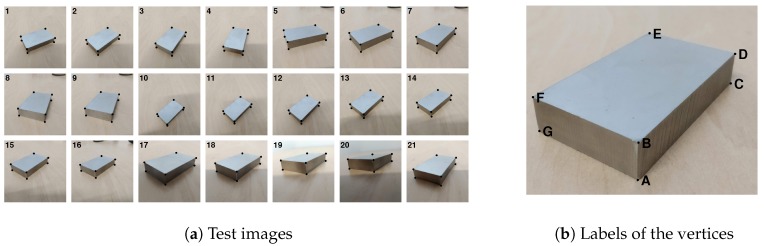
The 21 images (Photo Credits: *Copyright 2020 Baker Hughes Company. All rights reserved.*) of the metal block used for testing the DLT, Bouguet’s method (BOU), and weighted DLR (wDLT) algorithms. The control points (black dots) are manually selected on the images. They correspond to the seven visible vertices of the parallelepiped. The reprojection error, using DLT to calibrate the camera, is 2.5±1.3 pixels.

For the test, we used 21 images of a metal parallelepiped (see Figure 1a) whose size was 1 × 3 × 5 cm. The sizes of the blocks were accurate up to ±0.01 mm, but the edges and the vertices were blunt. The block stood on a table, lying on one of the two 3 × 5 cm faces.

The images were recorded varying the point of view with respect to the object. Each image had 3000 × 3000 pixels, and the same seven vertices of the block were visible, while the eighth was always hidden. Vertices were labeled with capital letters, as shown in Figure 1b.

The geometry of the object in the scene was known; thus, a 3D model of the block was generated. The seven visible vertices of the block were chosen as control points, because they were easy to recognize: from the 3D model of the object, the positions of the vertices were exactly known, while on the images, they were set manually. The DLT was applied: the reprojection error was 2.5±1.3 pixels (average ± STD), and the maximum error was 6.9 pixels for a single point. The average and the standard deviation of the reprojection error were first computed among points of the same image, and the results were averaged across different images. The average reprojection error for BOU was 2.5±1 pixels without distortion and 2.2±1.2 with second order radial distortion approximation. Adding more distortion parameters led to an ill conditioned estimation problem and numerical errors. The error of a few pixels on a high resolution image could be considered negligible, and the additional improvement obtained by including radial distortion was very slight. The two tests we performed are described below.

#### 2.2.1. Robustness to Random Error

**Figure 2:**
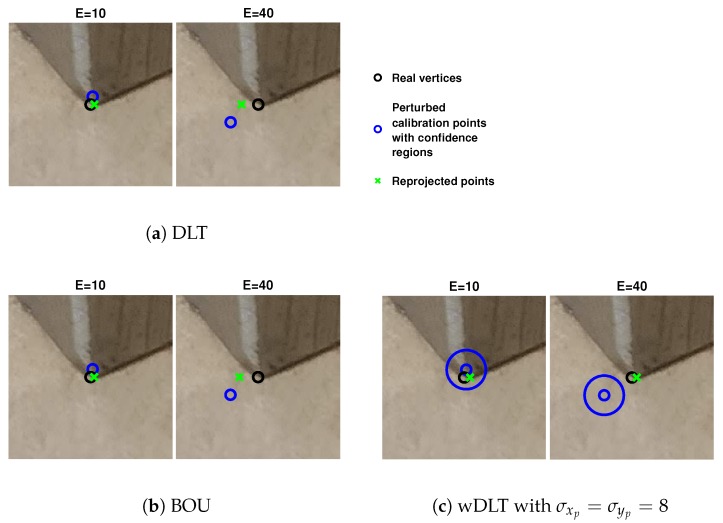
Example: Here, the calibration is performed on the seventh image (Photo Credits: *Copyright 2020 Baker Hughes Company. All rights reserved.*) with an error E on a control point of 10 and 40 pixels. The black circles are the unperturbed position of the control point, while in blue, we show the perturbed ones. In Figure 2c, the confidence region is shown as well: the radius of the circles is 24 pixels, corresponding to σ = 8. Here, wDLT can recover an error of 40 pixels on a single perturbed point, while DLT and BOU cannot.

In order to evaluate robustness to random errors, a first test was performed: the calibration was performed with DLT, BOU, and wDLT using perturbed control points. For each image $n_{E}$, control points of the image were randomly chosen, and an error, whose module was *E*, was added to these points in a random direction. Because of the randomness, the test was performed 10 times for each image, and $n_{E}=\left\{1,2,3\right\}$ and E={10,20,30,40}. For each point, the standard deviation was $\sigma_{x_{k}}=\sigma_{y_{k}}=1$, except for perturbed points, where $\sigma_{x_{p}}=\sigma_{y_{p}}=\left\{5,8,12\right\}$. In this test, the axes were equal, their rotation had no influence, and the confidence ellipses were circles. An example with $n_{E}=1$ and $\sigma_{x_{p}}=\sigma_{y_{p}}=8$ is shown in Figure 2: the real position of the vertices (unperturbed control points) is in black circles, and the perturbed ones are in blue. For wDLT, the confidence regions are shown as well. The green crosses are the reprojected points for DLT (Figure 2a), BOU (Figure 2b), and wDLT (Figure 2c). The reprojected points of the BOU algorithm, if no distortion was estimated, were very close to the DLT ones.

For each image, the average, the standard deviation, and the maximum of the reprojection error were computed. The reprojection error was the Euclidean distance on the image plane between the seven visible vertices and the control points without error.

#### 2.2.2. Occluded Vertices Scenario

The objective of the second test was to simulate a manual choice of the control points when reference points of the objects in the scene (such as corners) are occluded in the image. We performed the calibration without using some of the vertices’ positions in the images. Instead of using control points corresponding to the occluded vertices, the median points of the block edges were used in addition. The world points corresponding to the median were exactly known, while the corresponding points on the image were not. In order to have comparable results, each additional control point on the images was chosen systematically, using the following equation:

(7) $$\left[\begin{array}{c} u_{XY}\\ v_{XY} \end{array}\right]=\left[\begin{array}{c} u_{X}\\ v_{X} \end{array}\right]+m\left[\begin{array}{c} u_{Y}-u_{X}\\ v_{Y}-v_{X} \end{array}\right]$$

where $\left[u_{XY},v_{XY}\right]$ are the coordinates of the point along the edge XY, $\left[u_{X},v_{X}\right]$ and $\left[u_{Y},v_{Y}\right]$ are the coordinates of two vertices X and Y of the edge, and m∈[0,1] is an adimensional parameter. The coordinates of the control point $\left[u_{XY},v_{XY}\right]$ varied with *m* between the vertices along the edge XY.

Two scenarios were simulated:

1. Vertices C and D were occluded, and two additional control points were chosen on the edges AC and DE.
2. Vertices C, D, and E were occluded, and three additional control points were chosen on the edges AC, DE, and EF.

**Figure 3:**
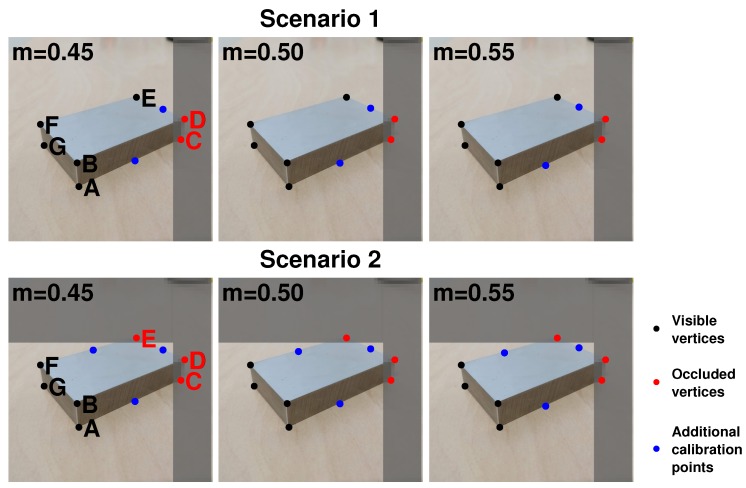
Scenarios with two and three occluded vertices: the black dots are the visible vertices, the red dots the occluded vertices (not used in calibration), and blue dots the additional points along the edges. The figures show the additional control points chosen along the edges with m={0.45,0.50,0.55}.

For each scenario and for each image, three sets of control points with m={0.45,0.50,0.55} were computed. Figure 3 shows, for both scenarios, the control points on the vertices in black, the occluded vertices in red, and in blue, the additional control points along the edge for m={0.45,0.50,0.55}. The reprojection error was the Euclidean distance between the real vertices and the reprojected vertices (i.e., without considering the error on the additional control points).

wDLT was tested with different values of uncertainty. For control points that were on vertices, the axis lengths of the confidence ellipses were the same ($l_{x_{i}}=l_{y_{i}}=1$). Instead, for control points that were not on vertices, but on edges, the axes of the confidence ellipses were set to be equal to one in the direction perpendicular to the edge ($l_{y_{i}}=1$) and equal to 3, 15, and $10^{6}$ in the direction parallel to the edge ($l_{x_{i}}=\left\{3,15,10^{6}\right\}$). We chose these values in order to evaluate the sensitivity of wDLT with different ellipse eccentricities to the estimation of the uncertainty σ.

**Figure 4:**
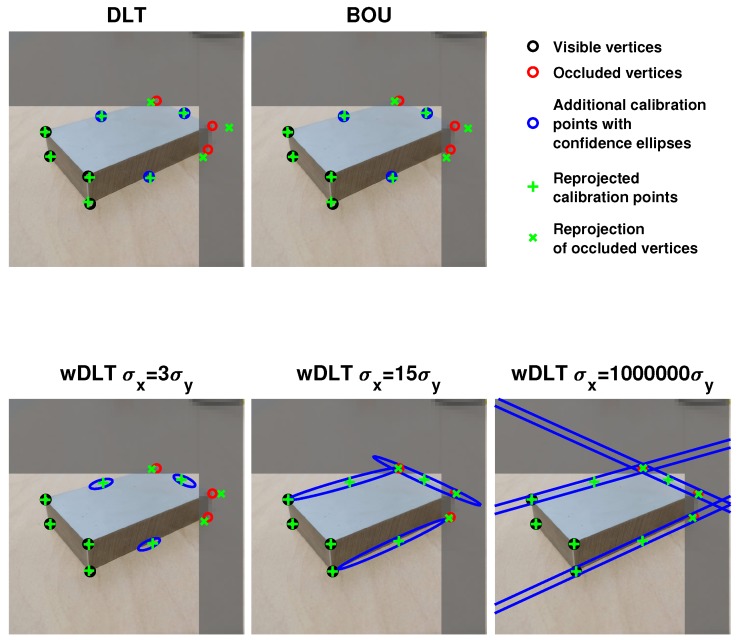
Scenario 2 with three occluded points and m = 0.50: black circles are the visible vertices, the red ones the occluded vertices (not used in calibration), and the blue ellipses the confidence ellipses of the additional control points on the edges. We represent each ellipse 50 times bigger in order to make them clearly visible. The green pluses and the green crosses are respectively the reprojected control points and the reprojected occluded vertices. While DLT and BOU reach good alignment of the control points (black and blue circles), the reprojection error on the occluded vertices is high. Instead, wDLT reaches a better calibration because the error on the occluded vertices’ reprojection is low.

Figure 4 shows for the second scenario and for different ratios $l_{x_{i}}/l_{y_{i}}$ the control points, the occluded vertices, and the confidence region of the additional control points. As an example, the reprojected control points and reprojected vertices are also shown. For the sake of clear representation, all the confidence ellipses had sizes 50 times bigger than the real ones.

## 3. Results

### 3.1. Robustness to Error

The first test aimed to evaluate the robustness of the three algorithms (DLT, BOU, and wDLT); thus, the reprojection error was calculated with control points perturbed with random errors and with different values of uncertainty for wDLT. We varied both the module of the error E and the number of points $n_{E}$ affected by the error E. BOU calibration was performed without distortion estimation, because not all the random configurations of control points led to a solution; this fact was due to the estimation problem, which became ill conditioned when data did not contain enough information.

**Figure 5:**
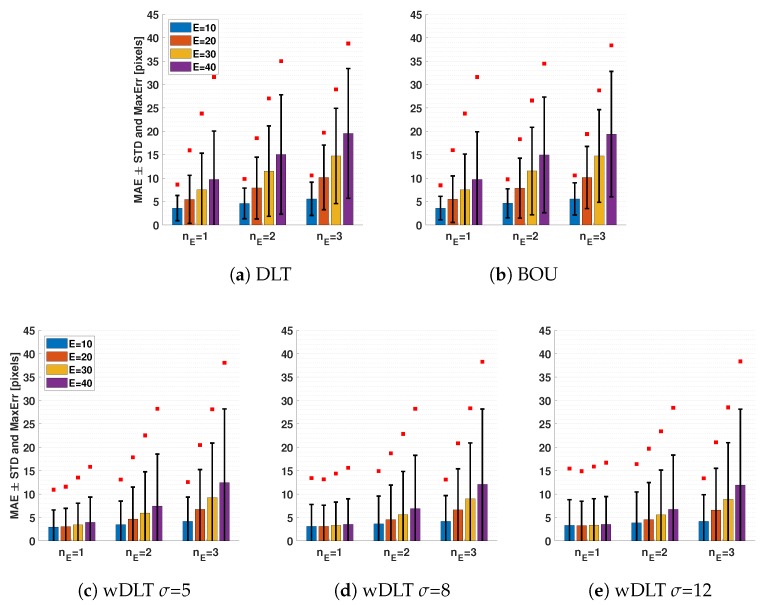
Reprojection error of the calibration with DLT, BOU, and wDLT with $n_{E}$ perturbed control points and error E; bars represent the mean absolute error (MAE), the black lines the average STD of the errors, and the red squares the average of the maximum error.

The reprojection error for random perturbations of control points (see Section 2.2.1) is summarized in the charts in Figure 5: vertical bars show the mean absolute error (MAE); the standard deviation (STD) is indicated by the black vertical lines, which show the ±1σ range; and red squares are the maximum absolute errors (MaxErr). The whole distributions of the error are shown in Appendix B.

As expected, the DLT and BOU performances decreases with high error E and number of errors $n_{E}$ (see Figure 5a). While the DLT and BOU algorithms only relied on the position of the control points, wDLT had the uncertainty of the points’ position. Because higher uncertainty was set for perturbed points, wDLT assigned less weight to the information about their position, while it focused more on the position of the others. In this way, the average error for each E and $n_{E}$ was lower for wDLT. Moreover, with wDLT, the MAE did not vary significantly when the values of uncertainties $\sigma_{x_{P}}$ and $\sigma_{y_{P}}$ of perturbed control points changed.

With only one perturbed point of seven control points ($n_{E}=1$), the error was almost fully recovered, and the performance was similar to the DLT with no perturbed points. Figure 2c shows an example where with wDLT, the reprojected point was almost unaffected by the position of the control point and was near the real vertex. For more than one of seven perturbed points, it was shown that the error was no longer recovered. The reason for that was the fact that the algorithms needed at least six control points, and with $n_{E}=1$ and high uncertainty, wDLT would consider only the remaining six low uncertainty points. On the contrary, with $n_{E}\geq 1$, less than six points were reliable; thus, wDLT needed the information of the perturbed points. However, wDLT would assign more weight to points with low uncertainty, and the average error would decrease as well.

It is worth noting that when the perturbation was small (E = 10), the maximum error did not improve with the exploitation of the uncertainty by wDLT. The reason was that the value of E was comparable with the uncertainty of the unperturbed points; thus, the best performance was achieved by setting in wDLT an equal uncertainty for all the points, which was equivalent to using DLT or BOU.

Summarizing: If the estimated uncertainty σ was too low for a subset of control points, the wDLT would take high error points more into account, and the MAE would increase because of the wrong information in input; if the uncertainty was too high, the algorithm would not consider important information from those points, and the MAE would increase as well. Therefore, in order to have better MAE than DLT and BOU, it was enough to set the uncertainty such that if a point was more uncertain than another one, the first had to have higher uncertainty than the second one, regardless of the exact ratio between the two uncertainties.

### 3.2. Occluded Vertices Scenario

**Figure 6:**
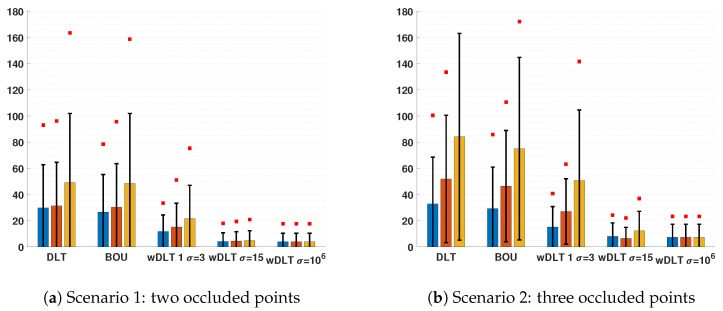
Reprojection error of the calibration with DLT, BOU, and wDLT with different settings of the uncertainty σ for points along the edges. The bars represent the average error, the black lines the average STD of the errors, and the red squares the average of the maximum error.

The results of the second test are shown in the charts in Figure 6. As for the first test, the BOU estimation of distortion parameters was not feasible; thus, they were not estimated. In both scenarios (with two or three occluded points), the algorithms showed the same behaviors, but the errors with three occluded points were always higher than the ones with only two occluded points, as expected.

The results with m=0.45 were better than the others because they corresponded to the points that were nearer to the real ones. Indeed, points with m=0.45 were chosen along the edge BD and nearer to D than to B, along the edge DE and nearer to E than to D, and along EF and nearer to E than to F; due to the perspective of the image, these points were nearer to the median of the edges, which was the point that was considered in the 3D model (see Section 2.2.2). For the same reason, the worst results were obtained with m=0.55. Figure 4 shows the reprojected vertices for the seventh image: both DLT and BOU minimized the error on control points (black and blue circles), but because blue circles had position errors, the alignment was wrong, which was clear by looking at the reprojection of occluded vertices. For wDLT, the confidence ellipses allowed the reprojected points to slide more along their major axes; thus, the results were better because the high variance along the edges produced a better estimation of the positions of the occluded vertices.

With a very high uncertainty set along the edge ($\sigma =10^{6}$), wDLT reached a similar MAE without depending on the position of the point along the edge (i.e., the values of m, according to our parametrization of its position). The reason was that with a very high σ along the direction of the edge, the resulting system of Equation (6) constrained the median points of the edge to be along the lines of each edge, but made them free to move along that. Thus, wDLT did not take into account if the positions of those points slid along the edges.

## 4. Discussion

The proposed method was a weighted DLT based algorithm, suitable when the uncertainty areas of the control points on the image were known or could be estimated. It did not require the knowledge of the exact values of uncertainty, because a gross estimation still improved the mean absolute error. To the best of our knowledge, no one has yet presented a calibration algorithm that exploits the anisotropic uncertainty of the control points by setting a weight matrix according to the lengths and rotation of the axis of uncertainty ellipses.

wDLT was also useful when there was no calibration pattern in the scene and the choice of the control points was manual. In this kind of situation, there were only a few control points: some of them corresponded to corners, and their uncertainty was low; others could be on edges, and their uncertainty was low only in the direction perpendicular to the edge; other points could be assigned in correspondence to a wide area, and their uncertainty was higher. If there were only a few corners, it was necessary to exploit as much information as possible about the points.

For infrared thermography in gas turbines, the image of the rotor blade was only partial and had low resolution. Many defects and artifacts were present on the image, and manual calibration was necessary. The lack of corners, reference points (the shape of the blade was smooth and curvy), and clear edges made the choice of control points very hard. Allowing the use of uncertainty ellipses (instead of single points), this step became easier and calibration more accurate.

Some works presented different calibration algorithms that could cope with outliers, but they required many points to be extracted. Tens of points are usually given from automatic feature extraction algorithms, and usually, many outliers are present. The presented results showed the advantages of using uncertainty information in the calibration problem. In the presented cases, the obtained error was lower compared to the DLT algorithm and Bouguet’s method.

The cost of this improvement was the knowledge of the uncertainty. In the first test, we supposed knowing the points with higher error. Huge improvement was reached only with $n_{E}=1$, but almost the same result could be reached applying the DLT to the six points without errors (which we supposed were known). For $n_{E}\geq 1$, there were only minor improvements.

The second test showed that the wDLT algorithm was useful when there were partially occluded objects with few visible corners. Introducing anisotropy in the uncertainty area of the edge points strongly improved the estimation of the positions of the hidden vertices.

The main advantage of this algorithm was that it offered the possibility of including anisotropic uncertainty information in the calibration, improving the solution. Even though this method did not provide a precise way to estimate the absolute uncertainty of the individual points, when these values were manually set, a strong improvement was observed as soon as the relative differences between uncertainties were assigned.

Infrared camera calibration for gas turbine thermography by using single control points and standard DLT led to gross and unacceptable errors in camera parameters, whereas by setting uncertainty areas, instead of control points, the camera could be properly calibrated by using weighted DLT.

## 5. Conclusions

We presented a method to set the weight matrix in a weighted Direct Linear Transformation (wDLT) algorithm, in order to take into account the anisotropic uncertainties of the positions of individual control points. Instead of control points, we proposed to use uncertainty ellipses with different axis lengths and angles. This feature became important when few control points were available. To demonstrate the effectiveness of the algorithms, we performed two tests: the first, to evaluate the robustness to random error, and the second, to simulate a scenario with few control points. We showed that wDLT with the uncertainty performed better (lower MAE) in both tests. In particular, in the second test, the calibration failed both with DLT and Bouguet’s method when several vertices were hidden, while wDLT still provided a successful calibration in these cases.

A suitable application of wDLT is in infrared thermography in gas turbines, where only a few control points can be chosen accurately, and then, manual selection of them is required. Accuracy in infrared camera calibration allows associating the correct temperature with the 3D model of the object in the field of view.

## Figures and Tables

**Figure A1:**
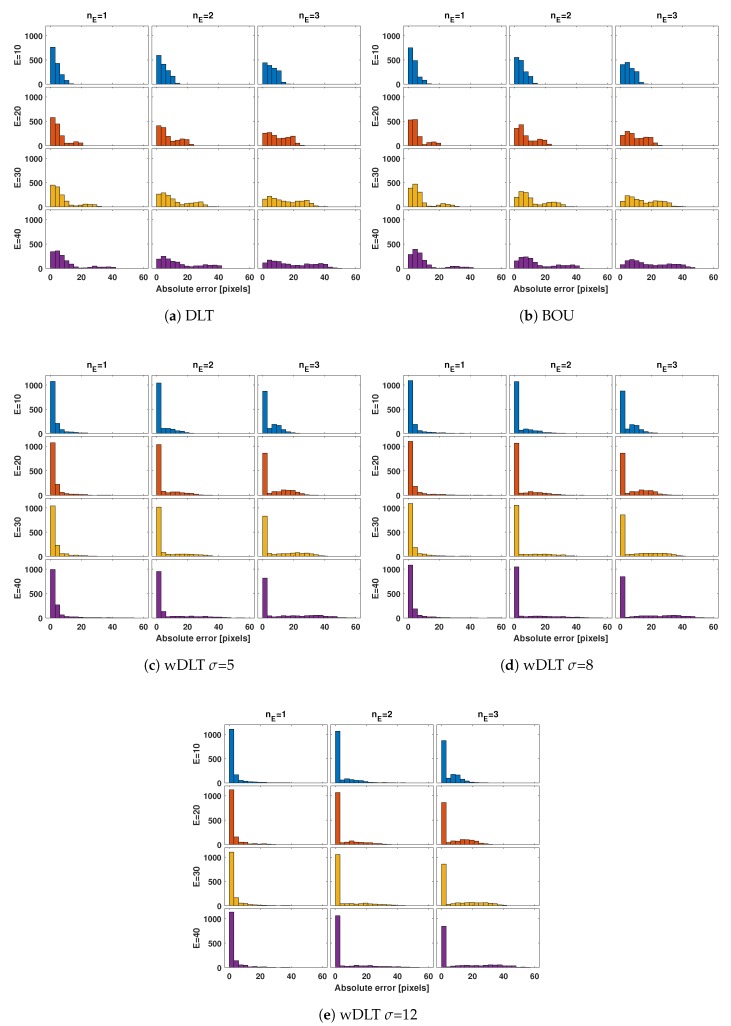
Distribution of the reprojection error of the calibration with DLT, BOU, and wDLT with $n_{E}$ perturbed control points and error E.

## Appendix A. Direct Linear Transformation

The Direct Linear Transformation (DLT) algorithm aims to solve the problem of determining the pinhole camera parameters from at least six correspondences between 2D image points and 3D world points. A camera model maps each point of the 3D world to a point of the 2D image through a projection operation. The pinhole camera model makes the assumption that the aperture size of the camera is small, so that it can be considered as a point. Thus, the ray of light has to pass across a single point, the camera center, and there are no lenses, no distortion, and an infinite depth of field.

The pinhole camera model is fully represented by a linear projection, which is expressed by the following equation:

(A1) $$\lambda {\hat{x}}_{i}={\mathrm{PX}}_{i}$$

where $X_{i}={\left[X_{i},Y_{i},Z_{i},1\right]}^{T}$ is the ith world point in homogeneous coordinates, ${\hat{x}}_{i}={\left[x_{i},y_{i},z_{i}\right]}^{T}$ is a 3D representation of the ith projected point, $P=\left[p_{ij}\right]\in {ℜ}^{3\times 4}$ is the projection matrix, or camera matrix, and λ∈ℜ is a free parameter. The parameter λ is usually set equal to the reciprocal of *z*, such as:

(A2) $${\tilde{x}}_{i}=\lambda {\hat{x}}_{i}=\frac{1}{z}{\hat{x}}_{i}={\left[\begin{array}{ccc} \frac{x_{i}}{z_{i}} & \frac{y_{i}}{z_{i}} & 1 \end{array}\right]}^{T}={\left[\begin{array}{ccc} u_{i} & v_{i} & 1 \end{array}\right]}^{T}={\left[\begin{array}{cc} x_{i}^{T} & 1 \end{array}\right]}^{T}$$

where ${\tilde{x}}_{i}$ is the ith projected point $x_{i}$ in the image plane in homogeneous coordinates.

Combining Equations (A1) and (A2), the coordinates of the image point $x_{i}$ are expressed by the following relationship:

(A3) $$x_{i}=\left[\begin{array}{c} u_{i}\\ v_{i} \end{array}\right]=\left[\begin{array}{c} x_{i}/z_{i}\\ y_{i}/z_{i} \end{array}\right]=\left[\begin{array}{c} p_{1}X_{i}/p_{3}X_{i}\\ p_{2}X_{i}/p_{3}X_{i} \end{array}\right]$$

where $p_{1}$, $p_{2}$, and $p_{3}$ are the rows of the projection matrix P. This relationship is not linear in the unknown parameters $p_{1}$, $p_{2}$ and $p_{3}$.

Through DLT, the nonlinear problem becomes linear in the unknown parameters, rearranging Equation (A3) as follows:

(A4) $$\left[\begin{array}{c} up_{3}X_{i}\\ vp_{3}X_{i} \end{array}\right]=\left[\begin{array}{c} p_{1}X_{i}\\ p_{2}X_{i} \end{array}\right]\Rightarrow \left[\begin{array}{c} -X_{i}^{T}p_{1}^{T}+0^{T}p_{2}^{T}+u_{i}X_{i}^{T}p_{3}^{T}\\ 0^{T}p_{1}^{T}-X_{i}^{T}p_{2}^{T}+v_{i}X_{i}^{T}p_{3}^{T} \end{array}\right]=\left[\begin{array}{ccc} -X_{i}^{T} & 0^{T} & u_{i}X_{i}^{T}\\ 0^{T} & -X_{i}^{T} & v_{i}X_{i}^{T} \end{array}\right]\left[\begin{array}{c} p_{1}^{T}\\ p_{2}^{T}\\ p_{3}^{T} \end{array}\right]=\left[\begin{array}{c} 0\\ 0 \end{array}\right]$$

These homogeneous equations can be solved linearly. Combining the equations for each point *i*, the homogeneous linear systems shown in Equation (3) is obtained.

## Appendix B. Error Distribution for Random Perturbation of Control Points

In this section, the error distributions for the test described in Section 2.2.1 are shown. Figure A1a,b show the error distribution using the DLT algorithm and Bouguet’s method, while in Figure A1c–e, we show the distributions for the wDLT algorithm with different σ settings for the perturbed points. Each bar chart represents the error distribution for different $n_{E}$ and E.
